# Effects of Mineral on Taxonomic and Functional Structures of Microbial Community in Tengchong Hot Springs via *in-situ* cultivation

**DOI:** 10.1186/s40793-023-00481-1

**Published:** 2023-03-22

**Authors:** Fangru Li, Weiguo Hou, Shang Wang, Yidi Zhang, Qing He, Wenhui Zhang, Hailiang Dong

**Affiliations:** 1 Center for Geomicrobiology and Biogeochemistry Research, State Key Laboratory of Biology and Environmental Geology, China University of Geosciences, Beijing, 100083 China; 2 CAS Key Laboratory of Environmental Biotechnology, Research Center for Eco-Environmental Sciences, Chinese Academy of Sciences (CAS), Beijing, 100085 China

**Keywords:** Mineral particles, Microbial communities, Taxonomic diversity, Functional groups, Stochastically adhesion

## Abstract

Diverse mineralogical compositions occur in hot spring sediments, but the impact of minerals on the diversity and structure of microbial communities remains poorly elucidated. In this study, different mineral particles with various chemistries (i.e., hematite, biotite, K-feldspar, quartz, muscovite, aragonite, serpentine, olivine, barite, apatite, and pyrite) were incubated for ten days in two Tengchong hot springs, one alkaline (pH ~ 8.34) with a high temperature (~ 82.8 °C) (Gumingquan, short as GMQ) and one acidic (pH ~ 3.63) with a relatively low temperature (~ 43.3 °C) (Wenguangting, short as WGT), to determine the impacts of minerals on the microbial communities taxonomic and functional diversities. Results showed that the mineral-associated bacterial taxa differed from those of the bulk sediment samples in the two hot springs. The relative abundance of *Proteobacteria*, *Euryarchaeota*, and *Acidobacteria* increased in all minerals, indicating that these microorganisms are apt to colonize on solid surfaces. The α-diversity indices of the microbial communities on the mineral surfaces in the WGT were higher than those from the bulk sediment samples (*p* < 0.05), which may be caused by the stochastically adhering process on the mineral surface during 10-day incubation, different from the microbial community in sediment which has experienced long-term environmental and ecological screening. Chemoheterotrophy increased with minerals incubation, which was high in most cultured minerals (the relative contents were 5.8 − 21.4%). Most notably, the sulfate respiration bacteria (mainly related to *Desulfobulbaceae* and *Syntrophaceae*) associated with aragonite in the acidic hot spring significantly differed from other minerals, possibly due to the pH buffering effect of aragonite providing more favorable conditions for their survival and proliferation. By comparison, aragonite cultured in the alkaline hot spring highly enriched denitrifying bacteria and may have promoted the nitrogen cycle within the system. Collectively, we speculated that diverse microbes stochastically adhered on the surface of minerals in the water flows, and the physicochemical properties of minerals drove the enrichment of certain microbial communities and functional groups during the short-term incubation. Taken together, these findings thereby provide novel insights into mechanisms of community assembly and element cycling in the terrestrial hydrothermal system associated with hot springs.

**Supplementary Information:**

The online version contains supplementary material available at 10.1186/s40793-023-00481-1.

## Introduction

Hot springs, as representatives of extreme environments, have been shown to have highly diversified and abundant microbial communities, which still exist many yet uncultured and unexamined microorganisms [1, 2]. These microorganisms may have evolved various metabolic systems to adapt to the local specific environment [3, 4], such as genes related to carbohydrate degradation of *Candidatus* Bathyarchaeia for thermal adaptation to terrestrial geothermal habitats [5] and genes related to light sensing and absorption of *Cyanobacteria* to cope with fluctuating environments [6]. A wide range of abiotic environmental factors has been proven to shape the hot spring microbial community structures and diversity, with pH and temperature considered the most critical factors [7–10]. Meanwhile, other geochemistry parameters exhibited underlying non-negligible effects, such as sulfate [11], total carbons [3], and critical metal elements (e.g., Ca^2+^, Mg^2+^, and Fe^2+^) [12, 13]. Hot spring sediments contain various minerals with high heterogeneity, where microbial diversity and responsiveness to environmental change were more significant than these of the water community [14]. Our previous study also demonstrated that sedimentary mineralogical compositions of hot springs were one of the major driving factors and generated special niches for specific microbial species [15].

More specifically, minerals provide nutrient resources (essential and trace elements), energy (electron donors or acceptors), and specific ecological niches/microhabitats to support the growth of microorganisms [16–18]. In nearly all ecosystems, minerals and microbes co-exist and co-evolve based on fundamental and preferential associations [19, 20]. A growing body of studies has demonstrated that minerals determine the microbial community assembly and colonization processes through field/lab experiments [21–23]. For instance, Whitman and colleagues found that bacteria and fungi colonized the surface across different mineral types (kaolinite, quartz, and ferrihydrite) within the same vegetation soil [24]. Specific microorganisms or microbial communities selectively attached to the surface of different minerals with varying intrinsic characteristics, such as mineral microtopography, chemical microenvironment (pH or Eh), surface charge, key nutrient contents, and weatherability [16, 19, 25]. Some minerals containing variable-valence elements can also affect microbial communities by generating energy through terminal electron acceptor or donor redox [26], especially iron-binding or sulfur-binding minerals [27, 28]. Wang et al. interpreted the bioavailability of metal sulfide minerals (mercury sulfides, pyrite, and marcasite) as electron donors that may be the key control on deep-sea chemosynthetic community activity and proliferation [29]. In addition, the influence of minerals on the community assemblage processes was also controlled by external factors (conditions of the bulk environments), such as land-use intensities [22], hot springs sedimentary composition [15], and forest plant cover [23].

Minerals also affect the biomass and diversity of microbial communities [19, 30, 31]. These effects can vary with mineral types. Wild and collaborators revealed that silicate minerals probes (labradorite and olivine) have high microbial richness and diversity than that of quartz and the soil samples in 9 months of field incubation [32]. The abundance of nutritive elements of incubated minerals was also proposed as an essential driver of mineral-associated biomass [24, 33]. In another study, a modest increase in bacterial biomass accompanied the increase observed in alpha diversity of microbial communities on minerals [22].

In addition, minerals impact the differential gene expression and functional groups of microbial community [34–36]. Many mineral elements (i.e., Cu, Mo, Fe, Ni, V, and P) contained in minerals act as critical factors [37, 38] and promote many aspects of microbial metabolisms and growth. For instance, the addition of ferrihydrite and hematite enhanced soil nitrogen-fixing activity and up-regulated transcription of *nifD* in *Anaeromyxobacter* and *Geobacter* [39]. The availability of Cu in borosilicate glass minerals influences CH_4_ oxidation rates of *Methylosinus trichosporium* OB3b and promotes the expression and patterns of soluble/particulate methane monooxygenases (sMMO/pMMO) [40, 41]. Moreover, several studies have revealed that rock/minerals environmental changes can cause intracellular reactions of microorganisms and affect the level of protein expression [42, 43]. Fe and Mg in volcanic rocks promoted cell division of *Cupriavidus metallidurans* CH34 and up-regulated phosphate limitation-related proteins [42]. While under iron-limiting conditions, basalt up-regulated expression of genes encoding putative components of ABC-type transporters, porins, and extra-cytoplasmic solute receptors in *Cupriavidus metallidurans* CH34 [44].

In the extreme environment, minerals act as a particular interface and additional selective pressure on microbes, due to their special physical properties and nutrient content [14, 15]. Such microbes’ selective colonization of different minerals has been observed in a subglacial environment and a submarine hydrothermal system [29, 33]. However, relatively few mineral colonization studies have been done in hot springs [15]. In this study, we elucidated how minerals influence hot spring microbial community composition and ecological functions through high-throughput sequencing. Microcosms with different mineral particles (hematite, biotite, K-feldspar, quartz, muscovite, aragonite, serpentine, olivine, barite, apatite, and pyrite) were cultured in two hot springs for 10-day. The objectives of this study were to: (i) demonstrate the effect of mineral addition on the hot spring microbial community and taxonomic structures, (ii) reveal the impacts of minerals on microbial diversity, and (iii) delineate the ecological function characteristics of mineral-associated microbial communities.

## Methods

### Sampling site description and experimental design

**Fig. 1:**
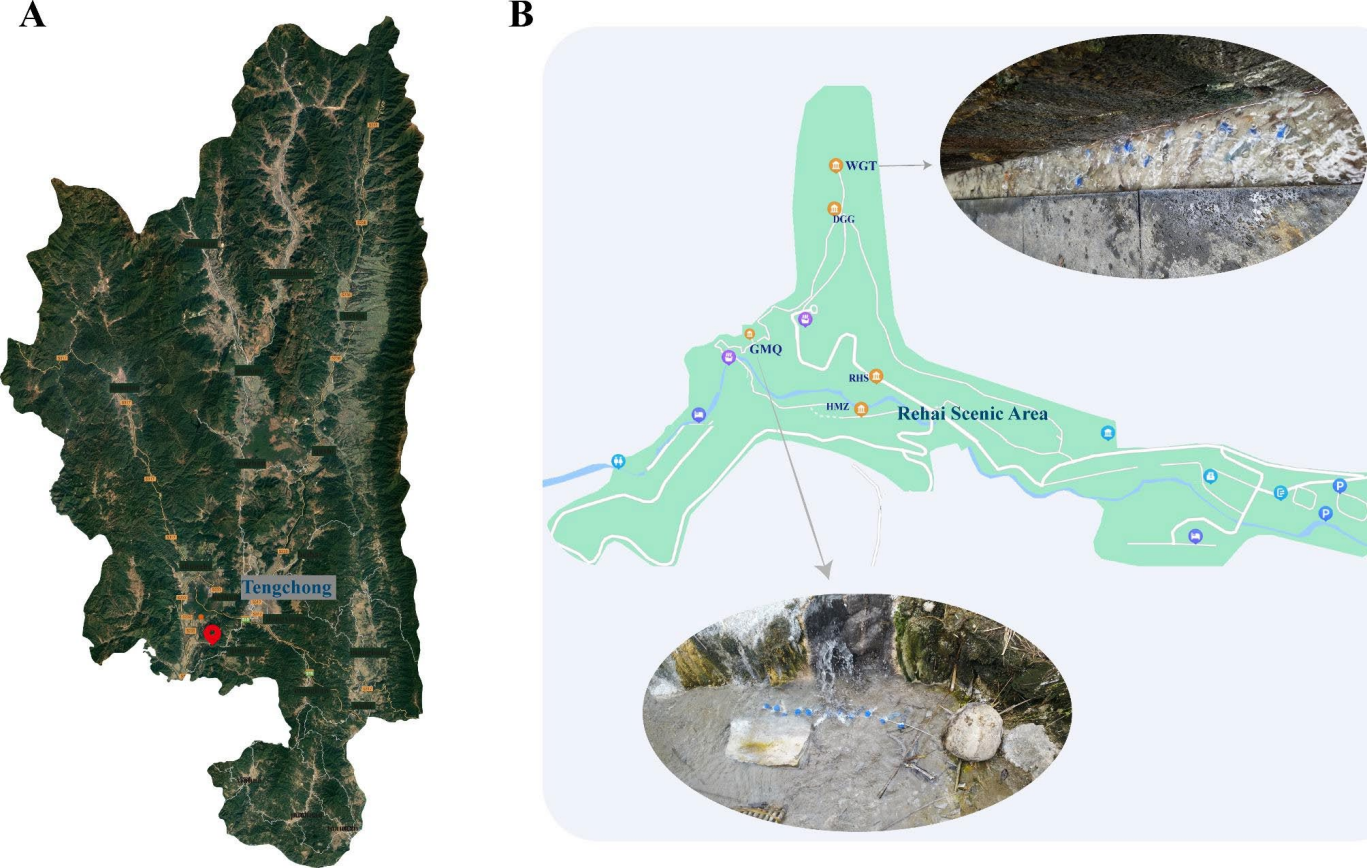
Overview of sampling sites and mineral microcosms. (A) sampling locations in Rehai National Geological Park, and (B) sampling locations in two hot springs (GMQ and WGT).

The study area is located in Rehai National Geological Park in Tengchong County, Baoshan City, Yunnan Province, China (Fig. 1). Two hot springs for mineral microcosm experiments were selected, which were obviously different in physical and chemical properties. Wenguangting (shorted as WGT) was an acidic spring (pH ~ 3.63) with a relatively low temperature (~ 43.3 °C) and a relatively small water flow. Gumingquan (shorted as GMQ) was an alkaline (pH ~ 8.34) spring with a high temperature (~ 82.8 °C) and a large water flow. The environmental conditions have been described in our previous studies [7, 15]. Before the microcosm experiment, water temperature and pH were measured with a portable meter (Hach, IA, USA). Sedimental samples in the middle of the spring courses were collected for X-ray diffraction (XRD) analysis. XRD was conducted with a Rigaku Smart Lab X-ray powder diffractometer with Cu K-alpha radiation (200 kV, 45 mA) with scanning angles from 3 to 70° two theta at a scanning speed of 2° per minute. The obtained data were analyzed by the software Jade 6 to identify minerals. Results showed that the mineralogical composition of the sediment mainly included quartz and kaolinite in WGT sediment, and quartz, albite, and K-feldspar in GMQ sediment.

Eleven minerals existed in the study area, i.e., hematite, biotite, K-feldspar, quartz, muscovite, aragonite, serpentine, olivine, barite, apatite, and pyrite obtained from https://www.bzwz.com/ accessed in January 2011, were ground (~ 1 cm ⋅ 1 cm ⋅ 1 cm), rinsed with 1 N HCl to remove adsorbed elements, washed with distilled water, and air-dried. Such particle size was used to facilitate the mineral collection, let the spring water flow through, and avoid solidification during incubation. About 20-g mineral particles were filled into sterilized 50-mL 16-gauge-syringe-needle perforated (with the diameter of the holes about 2 mm) polypropylene centrifugal tubes separately and incubated underwater in the two hot springs in September 2019. The microcosms were incubated in spring water for ten days. This incubating time was shortened comparing our last study [15] for 70 days to avoid much authigenic mineral precipitation in the microcosms. After incubating, mineral microcosms and *in-situ* sediment were collected in sterilized polypropylene tubes, frozen and transported in dry ice, and stored at − 80 ℃ in the laboratory until further microbial community analyses.

### DNA extraction, PCR amplification, and sequencing

Genomic DNAs were extracted from 10 g sediment and cultured minerals using the DNeasy PowerMax Soil Kit (Qiagen, Carlsbad, CA, USA) according to the modified manufacturer’s protocol [45]. Overall, the DNA extraction steps were optimized by using three cycles of freeze-thaw, cell lysis, DNA concentration, and DNA purification with the mineral samples to improve the quality and yield of DNA. The V4 region of prokaryotic 16S rRNA was amplified with primer pair 515F (5’-GTGYCAGCMGCCGCGGTAA-3’) − 806R (5’-GGACTACNVGGGTWTCTAAT-3’) containing 12-bp unique barcode sequences at both 5’ ends [46]. The PCR amplification was conducted in 25-µL mixtures containing 1-µL DNA template (5 ~ 15 ng), 2-µL dNTP, 2-µL 10x PCR buffer, 2-µL of forward/reverse primers, 0.3-µL Takara Ex Taq HS DNA polymerase, and DNase-RNase-Free deionized water to adjust the volume. The PCR procedure consisted of 98 °C denaturing for 30 s, followed by 32 cycles of 98 °C for 10 s, 54 °C for 30 s, 72 °C for 45 s; and a final step of 72 °C extensions for 10 min. Subsequently, all the products were purified and pooled together with an equal molar amount from each sample for sequencing on the Illumina Hiseq platform. Sequence data have been deposited in the Genome Sequence Archive in the National Genomics Data Center, Beijing Institute of Genomics (China National Center for Bioinformation), Chinese Academy of Sciences, under accession number (CRA009208) that are publicly accessible at https://bigd.big.ac.cn/gsa.

### Sequencing data analysis

The 16 S rRNA gene sequence data were analyzed via an accessible Galaxy Pipeline (http://mem.rcees.ac.cn:8080) [47]. Briefly, the raw sequences were demultiplexed according to unique barcodes with one mismatch allowed and trimmed separately forward and reverse primer. Paired sequences were combined by using FLASH [48], and the sequences with an average quality score lower than 20 and sequence length lower than 140 bp were filtered by Btrim [49]. The sequences containing any ambiguous bases were deleted, and only sequences within 245–260 bp were kept. Subsequently, OTUs (Operational Taxonomic Units) were generated at a 97% sequence similarity threshold by using UPARSE [50]. Taxonomic assignment for representative sequences of each OTU was carried out via the Ribosomal Database Project classifier (RDP) (Wang et al., 2007) based on the SILVA database (version 138.1) [51]. To eliminate the influence of the difference in sequencing depth on downstream analyses, 138,990 reads were randomly resampled for each sample.

The taxonomy richness (Observed richness), evenness (Pielou_evenness), and diversity (Shannon index and inverse of the Simpson index (InvSimpson)) of the microbial communities were calculated based on the resampled OTU table. PCoA (Principal Coordinate Analysis) and NMDS (non-metric multidimensional scaling) based on Bray-Curtis distance were used to assess how the beta-diversity could be influenced by the prokaryotic community dispersion via a microbiome analyzing platform (https://www.microbiomeanalyst.ca/) [52]. Adonis and ANOISM tests were conducted to calculate the significance of the results. Hierarchical clustering was performed by PAST to simplify the differences of microbial communities among the different mineral microcosms, as well as the surrounding sediment samples, with an unweighted pair-group method with arithmetic means (UPGMA) based on Bray–Curtis dissimilarity index [53]. In addition, the linear discriminant analysis (LDA) of effect size (LEfSe) analysis was conducted to identify the mineral-associated microbial taxa differentially represented. The differences in the taxa at the phylum and genus levels with a logarithmic LDA score > 4 and a *p*-value < 0.05 were considered. Furthermore, functional Annotation of Prokaryotic Taxa (FAPROTAX) based on 16 S rRNA gene sequencing via Galaxy Pipeline was performed to predict the microbial potential metabolic functions (e.g., carbon metabolisms, nitrogen metabolisms, sulfur metabolisms, and energy source) in different mineral microcosms [54]. To recognize functional groups that showed significant differences in abundance between two groups among mineral microcosms, as well as the surrounding sediment samples, the bar plots were performed based on Welch’s t-test with Storey FDP multiple test correction within the STAMP software [55]. Construction of the extended error bar charts shows the average proportions together with differences between ratios in two communities and a 95% confidence interval when the *p*-value < 0.05 and effect size > 0.8%.

## Result

### Taxonomic structures of the mineral-associated microbial communities

**Fig. 2:**
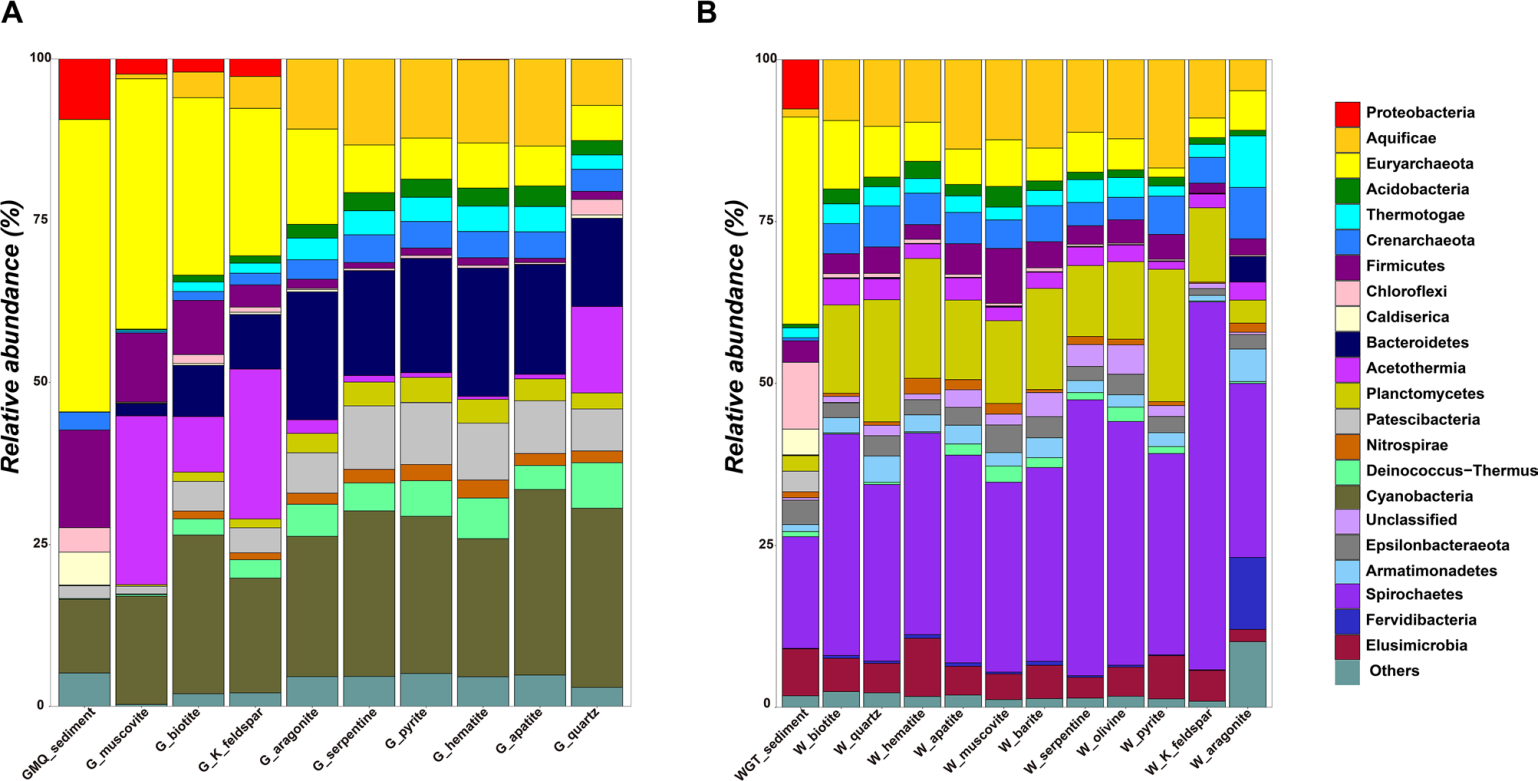
Taxonomic composition and relative abundance of the dominant phyla on the different minerals and the surrounding sediment samples. The other minor populations (rarer taxa with relative abundance < 2%) were summed as “Others” at the plot. G, GMQ; W, WGT.

**Fig. 3:**
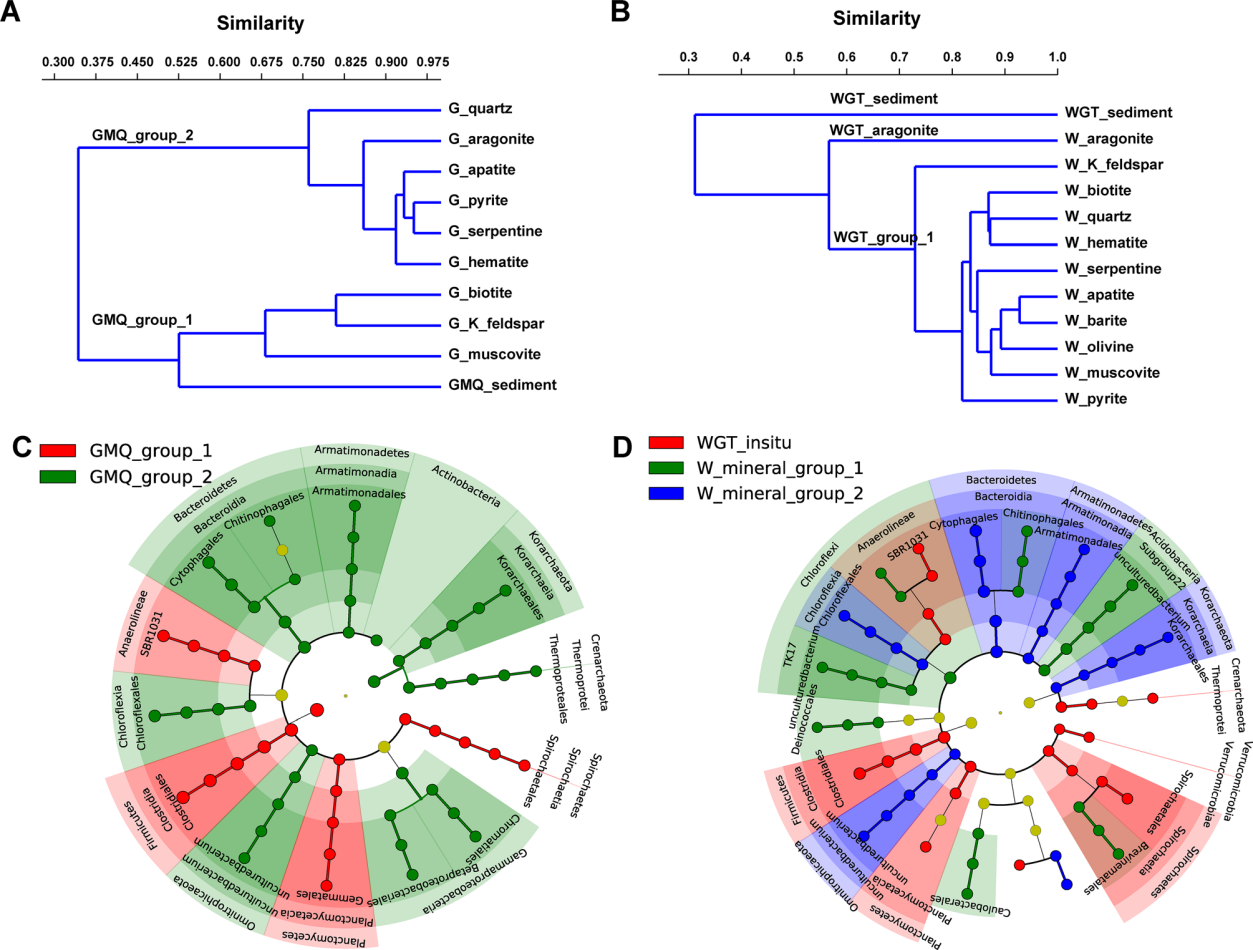
Hierarchical clustering and cladogram show significant differences between GMQ (A, C) and WGT (B, D) surrounding sediment samples and mineral microcosms. The sub-clusters separated in the clustering trees were identified as “GMQ_group_1”, “GMQ_group_2”, “WGT_sediment”, “WGT_aragonite”, and “WGT_group_1” groups. Colored dots represent the taxa with significant differences in abundance between different successions, and cladogram circles represent the phylogenetic taxa from phylum to order. The yellow node stands for shared features. Only the LDA score > 4 was shown. G, GMQ; W, WGT.

Overall, microbial communities from different mineral microcosms in the two hot springs were evidently distinguished from each other (Figs. 2 and 3). In GMQ, the surrounding sedimentary communities were dominated by *Aquificae* (~ 45.1%), *Crenarchaeota* (~ 15.1%), *Proteobacteria* (~ 11.4%), *Acetothermia* (~ 9.3%), and *Thermotogae* (~ 5.2%). In the microcosms with biotite, K-feldspar, and muscovite (Fig. 2A), the most dominant bacterial phylum was *Aquificae* (22.8-38.7%), followed by *Proteobacteria* (16.7-24.5%), *Firmicutes* (8.6-26.1%), and *Crenarchaeota* (8.4-10.6%). The dominant taxa phyla on K-feldspar were *Firmicutes* (~ 23.1%), *Aquificae* (~ 22.8%), *Proteobacteria* (~ 17.7%), and *Euryarchaeota* (~ 8.5%). With respect to other minerals, *Proteobacteria* ranked the highest relative abundances (21.3-28.7%), which was followed by members of *Euryarchaeota* (16.2-19.8%), *Acidobacteria* (10.8-13.5%), and *Aquificae* (6.2-14.7%) in the microcosms with hematite, apatite, pyrite, aragonite, and serpentine. *Proteobacteria* (~ 27.7%) also ranked the highest relative abundance in the quartz microcosm, followed by *Euryarchaeota*, *Firmicutes*, *Acidobacteria*, and *Planctomycetes* (Fig. 2A).

In WGT, sequencing results showed that dominant phyla in sediment mostly consisted of *Aquificae* (~ 32.0%), *Proteobacteria* (~ 17.2%), *Crenarchaeota* (~ 10.2%), *Acetothermia* (~ 7.6%), and *Thermotogae* (~ 7.2%). While the *Proteobacteria* had the highest relative abundances across all mineral microcosms in WGT. 10 out of 11 mineral microcosms (WGT_group_1 in Fig. 3B) were characterized by a high proportion of phylum *Euryarchaeota* (11.5-20.4%) and *Acidobacteria* (9.0-16.7%). The following microbial members were *Thermotogae* (4.8-9.0%) in hematite, pyrite, and K-feldspar, Aquificae (4.8-10.6%) in apatite serpentine, olivine, biotite, and quartz, *Chloroflexi* (~ 8.5%) in muscovite, and *Caldiserica* (~ 4.4%) on barite. Notably, the aragonite microcosm was dominated by *Proteobacteria* (~ 26.8%), with *Spirochaetes* being the second most abundant phylum (~ 11.1%), followed by unclassified species (~ 10.2%), *Bacteroidetes* (~ 8.0%), and *Caldiserica* (~ 7.9%) (Fig. 2B).

The clustering results based on the microbial community distances could divide the microbial communities in mineral microcosms and surrounding sediment samples into two groups (GMQ_group_1 and GMQ_group_2) for GMQ, and three groups (WGT_sediment, WGT_aragonite, and WGT_group_1) for WGT (Fig. 3A and B). LEfSe analysis further revealed the significantly different taxa at phylum to genus levels explaining the observed difference in microbial communities across different groups (Fig. 3C and D). A total of 11 phylotypes at the class level were discovered as high-dimensional biomarkers for separating the GMQ_group_1 and GMQ_group_2 microbial community in GMQ. Members from *Thermoprotei*, *Chloroflexia*, *Bacteroidia*, *Armatimonadia*, *Korarchaeia*, *Gammaproteobacteria*, and the uncultured bacterium belonging to phylum *Omnitrophicaeota* were the key microbial taxa in GMQ_group_2 that differentiated from the microbial communities in GMQ_group_1 (Fig. 3C). A total of 13 phylotypes at the class level were discovered as high-dimensional biomarkers for separating the three groups of microbial communities in WGT. Microbial members from TK17, Subgroup22, the order of *Deinococcales*, and *Caulobacterles* were responsible for differentiating the community in the WGT_aragonite microcosm from those in other samples. Whereas *Chloroflexia*, *Bacterodia*, *Armatimonadia*, *Korarchaeia*, and uncultured bacterium belonging to the phylum *Omnitrophicaeota* distinguished WGT_group_1 from other samples (Fig. 3D). Notably, mineral microcosms enriched high content of unclassified bacterium at family and genus levels.

### Taxonomic diversity of the mineral-associated microbial communities compared to the surrounding bulk sediment samples

**Fig. 4:**
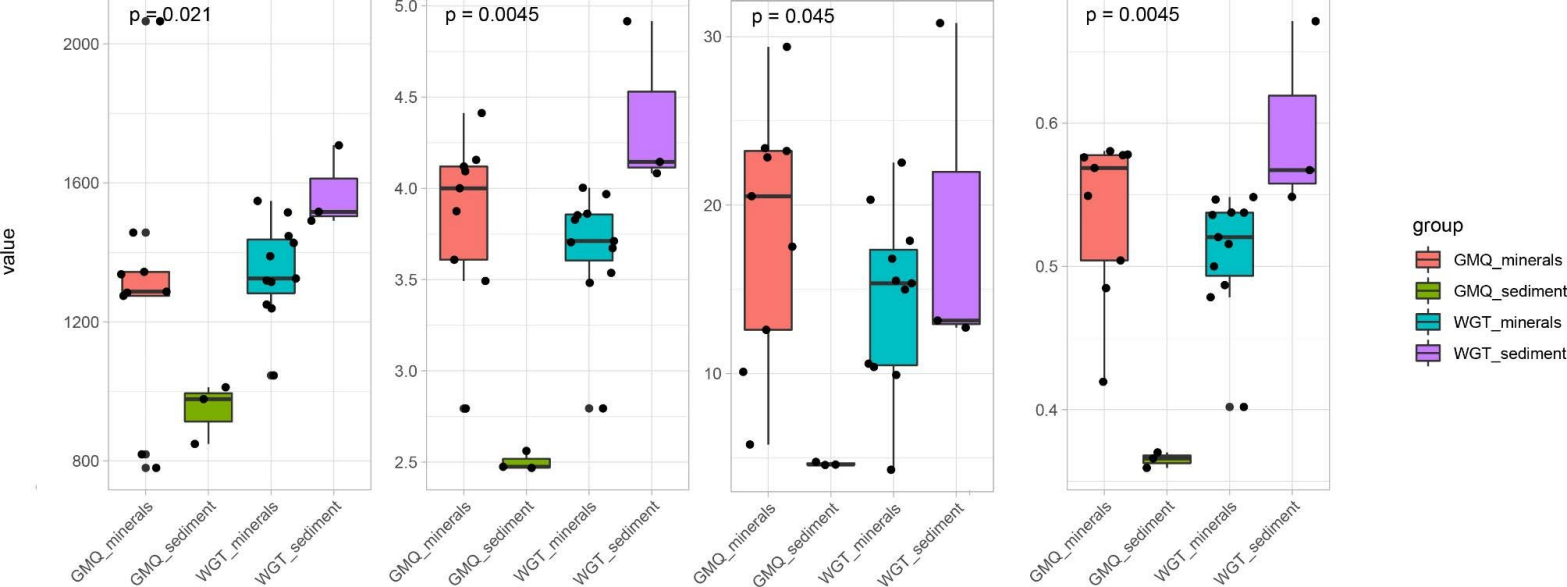
Comparison of alpha diversity measures of microbial communities at different mineral microcosms in two hot springs. Pairwise t-tests were performed for each pair of comparisons, GMQ_sediment vs. GMQ_minerals and WGT_sediment vs. WGT_mineral, at each OTU level to determine significance (*P* < 0.05)

The α-diversity was estimated based on observed richness, Pielou_evenness, Shannon, and InvSimpson indices to reflect the community diversity and species richness of each sample (Fig. 4). The overall richness and evenness of the microbial community in the GMQ mineral microcosms were significantly higher than those in the surrounding sediment (*p* < 0.05), which indicated that mineral microcosms greatly enhanced the community alpha diversity in GMQ. In contrast, WGT sediment microbial communities were significantly more diverse than those on the cultured minerals (*p* < 0.05). Noteworthily, the community α-diversity of GMQ surrounding sediments was the lowest among all the samples, and the differences between minerals and surrounding sediment in GMQ were more conspicuous than WGT. The alpha diversity of microbial communities in different minerals did not show significant differences in the two hot springs (*p* > 0.05).

**Fig. 5:**
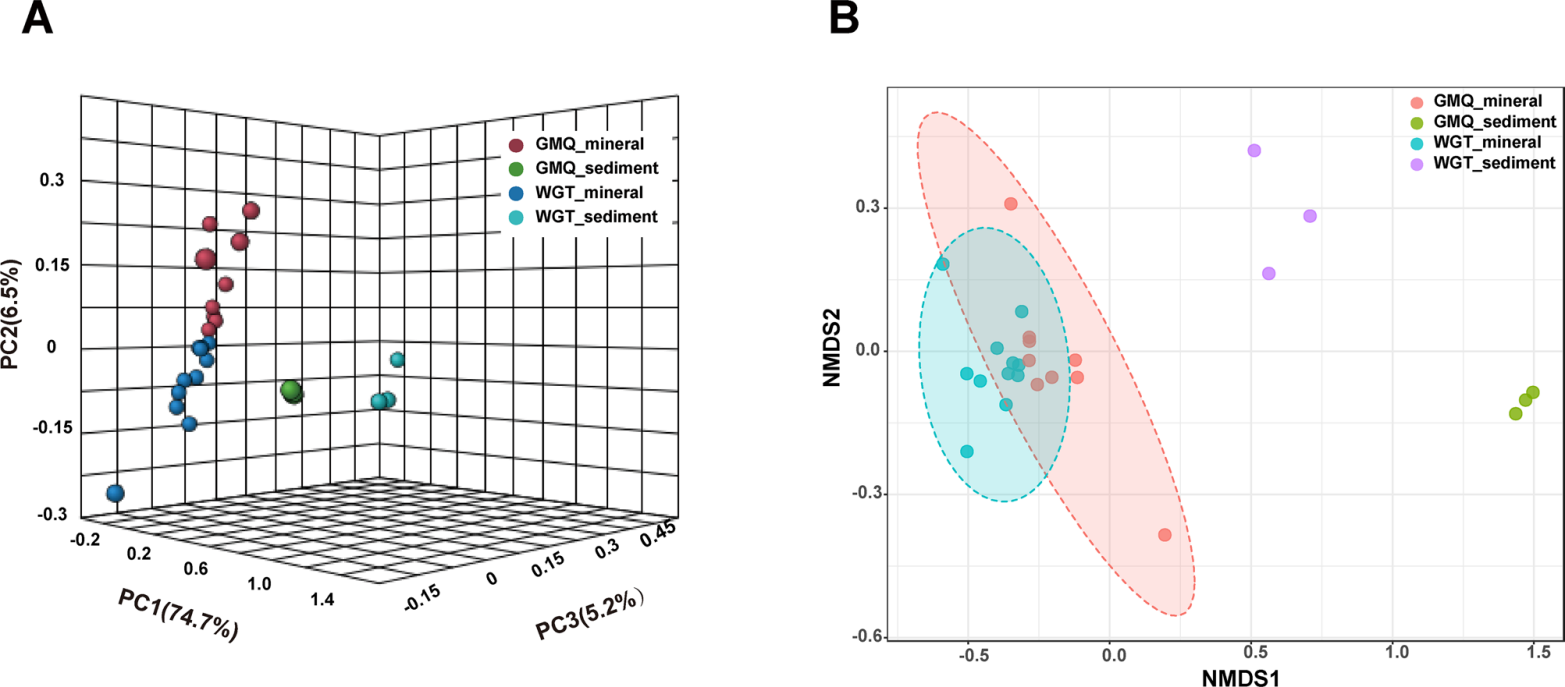
Three-dimensional principal coordinate analysis (PCoA) and non-metric multidimensional scaling (NMDS) of feature level based on the Bray–Curtis dissimilarity. Points situated closer together on the plot represent similar communities

Then, we carried out PCoA and NMDS analyses and dissimilarity tests (PERMANOVA and ANOSIM) to evaluate the beta-diversity differences between minerals and surrounding sediment samples of the microbial communities. PCoA plot based on Bray-Curtis’s distance metrics showed that bacterial community structure (at the feature level) of minerals and surrounding sediment samples were well separated. The three main axes of PCoA explained 81.2% of the variation, indicating that they could represent the characteristics of the microbial community’s composition, of which 74.7% variation was explained by PC1, 6.5% variation was explained by PC2, and 5.2% variation was explained by PC3, respectively (Fig. 5A). The differences between the microbial communities in mineral microcosms and surrounding sediments from the two springs were also revealed by the NMDS dissimilarity test based on Bray–Curtis’s distance (*p* < 0.001) (Fig. 5B).

### Predicted ecological function of microbial communities by FAPROTAX analysis

**Fig. 6:**
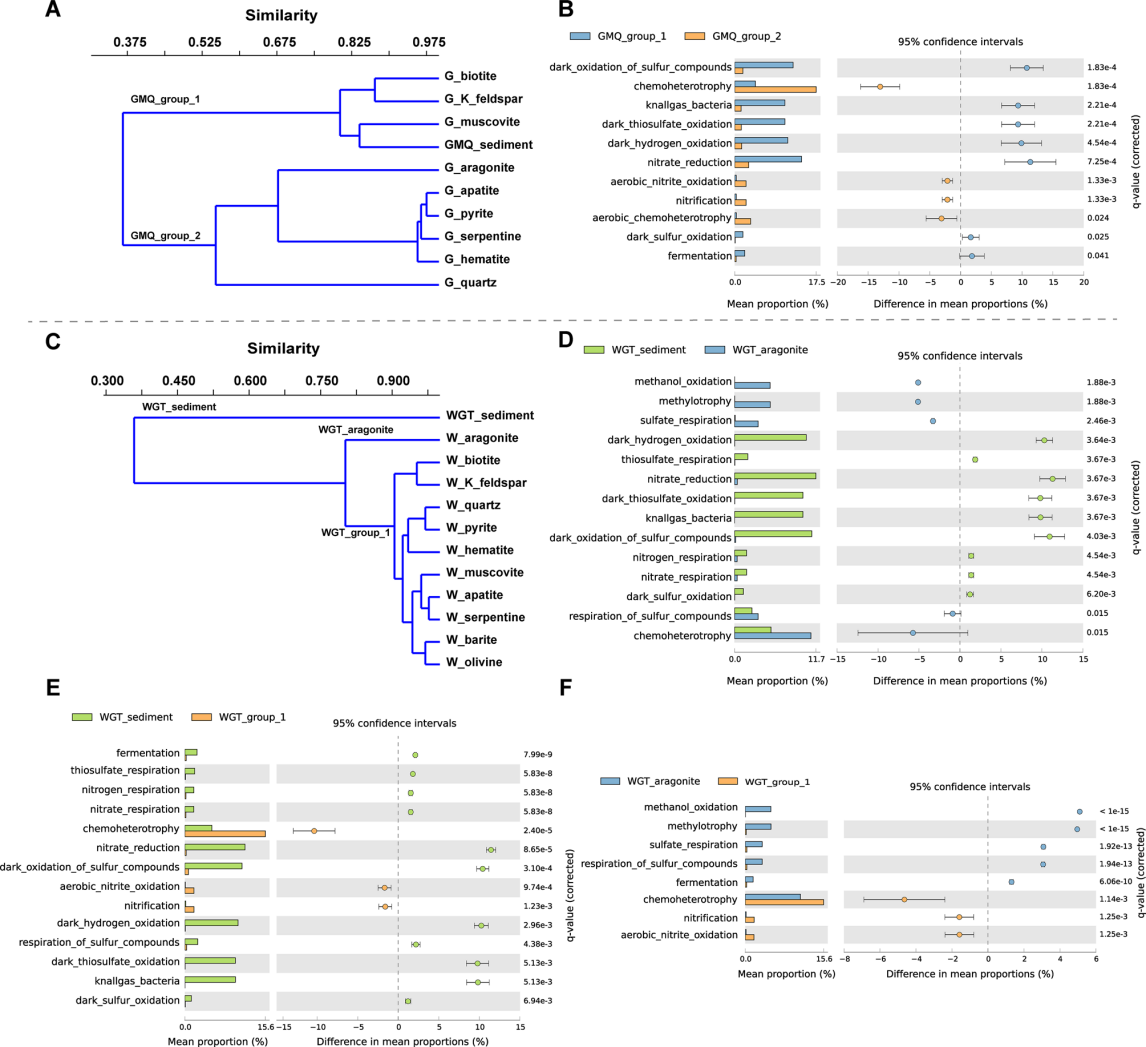
Hierarchical clustering of predicted microbial functions based on the FAPROTAX database in two hot springs (A, C). Bar plot of significantly different microbial functions in two groups obtained by two-sided Welch’s t-test with Storey FDR multiple test correction (p-value < 0.05 and effect size > 0.8%) (B, D, E, and F)

A total of 52 microbial community functional groups were annotated based on 2377 out of 10,791 OTUs, which mainly involved energy sources, as well as C, N, and S metabolism in these studied samples (Figure S1). In parallel to the microbial community compositions, microbial metabolic potentials varied among the different cultured minerals. The metabolic diversity of surrounding sediment was relatively higher than minerals in two hot springs based on the Shannon, Simpson, and InvSimpson indices (Table S1). Similar to the microbial community composition groupings, the metabolic functions of cultured minerals and sediment samples can also be divided into two groups in GMQ (GMQ_group_1 vs. GMQ_group_2) and three groups in WGT (WGT_sediment vs. WGT_aragonite vs. WGT_group_1) by hierarchical clustering, respectively (Fig. 6A, B). Similar to surrounding sediment samples of GMQ, the dominant functions from the microcosms with biotite, K-feldspar, and muscovite (GMQ_group_1) were related to C-cycles (fermentation), H-cycles (i.e., knallgas bacteria and dark hydrogen oxidation), S-cycles (i.e., dark oxidation of sulfur compounds, dark thiosulfate oxidation, and dark sulfur oxidation), and N-cycles (i.e., nitrate reduction). Chemoheterotrophy across hematite, quartz, aragonite, apatite, serpentine, and pyrite microcosms occupied 14.7-20.5% in relative abundance. In the GMQ_group_2 microcosms (quartz, apatite, pyrite, serpentine, and hematite), functional groups were mostly associated with N-cycles (i.e., nitrification and aerobic nitrite oxidation), chemoheterotrophy, and aerobic chemoheterotrophy compared to GMQ_group_1 (Fig. 6A, B). Moreover, an UpSet plot showed that the unique ecological function of cultured hematite was anoxygenic photoautotrophy Fe oxidizing and anoxygenic photoautotrophy H_2_ oxidizing.

Hierarchical clustering showed that the predicted functions of the surrounding sediment samples were significantly different from mineral microcosms in WGT (Fig. 6C). All mineral microcosms enriched the functions related to chemoheterotrophy (11.0-21.4%), depleted in H-cycles (i.e., knallgas bacteria and dark hydrogen oxidation), S-cycles (i.e., thiosulfate respiration, dark sulfur oxidation, dark thiosulfate oxidation, and dark oxidation of sulfur compounds), and N-cycles (i.e., nitrate reduction and nitrate respiration), by comparing with the surrounding sediment (Fig. 6D). In the WGT_group_1, functional groups were high in N-cycles (i.e., aerobic nitrite oxidation and nitrification) and chemoheterotrophy by comparing with surrounding sediment samples (Fig. 6E). Nevertheless, the enriched functional groups in aragonites were mainly related to C-cycles (i.e., methylotrophy and methanol oxidation) and S-cycles (i.e., respiration of sulfur compounds and sulfate respiration) compared to the surrounding sediment samples and WGT_group_1 (Fig. 6F).

## Discussion

### Minerals’ influence on the microbial community composition and diversity

Microcosms with minerals were set up and cultivated in hot springs to determine the role of minerals in the microbial community. To make the geochemical consistency of the water between the inside and outside of the mineral microcosms, the tubes were perforated on their bodies. 16 S rRNA gene sequencing analyses revealed that the taxonomic composition, diversity, and function of bacterial communities in hot springs were affected by different minerals. Such selective effect and colonization of minerals/rocks have now been evidenced in several ecosystems (forest soil, ocean, deep-formation, etc.). Specific microbial communities colonizing on minerals, such as goethite, illite [56], silicate minerals (labradorite and olivine) [32], apatite [57–59], obsidian [59, 60], and biotite [57], substantially differed from those in the *in-situ* soils. In our study, the presence of cultured minerals in hot springs has been demonstrated to alter the relative abundances of different microbes, which indicates a selective process of community assembly. Indeed, microbial communities uniquely adapt to their mineralogical environments largely due to the physicochemical properties and specific structure of different minerals. For example, mineral microhabitats (mica, basalt, and rock phosphate) contained nutritive elements such as P, K, Na, and Mg, which have been shown to determine and select bacterial communities with distinct structures [61]. At the same time, mineral weatherability has a prominent impact on microbial communities and can stimulate the growth of effective-weathering bacteria (e.g., β-Proteobacteria) on their surface [58, 59, 62, 63]. *Proteobacteria* and *Bacteroidetes*, as the dominant bacterial phyla in terrestrial and aquatic habitats, are positively related to organic matter [64] and have been detected on minerals [58, 59, 65]. Our previous research has also confirmed that the phyla *Aquificae*, *Proteobacteria*, *Firmicutes*, *Deinococcus-Thermus*, and *Bacteroidetes* were abundant in GMQ and WGT water [14]. Similarly, the relative abundance of Proteobacteria on the surface of minerals enriched by comparing with those of the surrounding sediment samples in both hot springs, indicating that they are apt to colonize and inhabit mineral surfaces. In contrast, *Aquificae* and *Crenarchaeota* were also shown to be less abundant on minerals than those in surrounding sediments, which suggests less competitiveness or physicochemical conditions for attachment than other microbes. The high proportion of uncultured bacteria within mineral microcosms confirmed our previous speculation that minerals could be used as a potential substrate for enriching microbial dark matter in hot springs [15]. There were also great differences in the response of microorganisms to minerals in different hot springs.

As the clustering results showed, the hot spring sedimentary mineralogical environment determined the microbial community’s similarity with these associated with the cultivated minerals. The bulk sediments of the GMQ pool were mainly composed of quartz, feldspar (albite, K-feldspar, and microcline), smectite, and biotite [15]. Accordingly, the enriched microbial communities on the surface of biotite, K-feldspar, and muscovite microcosms (K-bearing silicate minerals) were similar to those in surrounding sediments in our study (GMQ_group_1 in Fig. 3A). Quartz and kaolinite dominated WGT sedimentary mineralogical compositions. Conversely, microbial communities of the WGT spring did not cluster according to the massive sediment environment. Mineral microcosms were clustered into two completely different groups (i.e., aragonite and group_1 in Fig. 3B), which were quite different from the surrounding sediments (overall average dissimilarity > 66.9). As mentioned by Wild and collaborators, this result may reflect more dynamic conditions with respect to physicochemical conditions and microbial diversity [32]. The more dynamic environment in the WGT spring may delay the interaction between microorganisms and minerals. Additionally, kaolinite is clay with fine particles in water. It could make up a special micro-niche with small matrix pores, in which the water and solutes spread at a slow rate. So, we speculate that the existence of kaolinite was the main reason for the difference in the microbial communities between the sediments and the mineral microcosms.

Additionally, the diversity of microbial communities was impacted differently by minerals, which may also be related to the physicochemical characteristics of hot springs. According to our previous research, mineral heterogeneity in sediments had a positive impact on the alpha diversity of hot spring microorganisms; different minerals favored different species may ensure the high microbial diversity in hot spring sediments [14, 15], which is consistent with the WGT mineral microcosms where lower community diversities than that of surrounding sediment were observed in this study. Minerals can provide energy (via electron donors or acceptors), nutrients, or microhabitat for microorganisms, which affect the distribution of hot spring microorganisms in different minerals, as revealed in our study. Although there was more prominent heterogeneity in *in-situ* sediment, we speculated that the microbial community stochastically adheres to the surface of fresh minerals with the water flow in a short time of cultivation, and diverse microorganisms simultaneously compete for favorable sites. Fresh mineral particles can enrich more species because there are no dominating taxa to monopolize energy and nutrient resources, different from the microbial community in sediment, which has experienced long-term environmental and ecological screening. This can better explain the little diversity difference between WGT minerals and surrounding sediments. On the contrary, the pure mineral particles after 10-day incubation significantly harbored a high microbial diversity on their surface than that of sediment samples in GMQ. A possible explanation for this might be that minerals provide additional spaces and microenvironments for thermophiles to grow/inhabit on their surfaces in the withstand high-temperature alkaline environment.

### Response of hot spring microbial community ecological function to minerals

Similar to the community structure, community ecological functions were controlled by different minerals. Different microbial taxa may have different functions, and different functional groups are often filtered into different environments that characterize biochemical cycles in niches [66, 67]. Mounting evidence has demonstrated that minerals can stimulate or inhibit the metabolic activity of microorganisms attaching to the surfaces [68, 69], and induce the expression of different genes [42, 43]. Consistent with these studies, our findings suggest that mineral types could strongly shape the microbial functional structure. Chemoheterotrophy increased with mineral incubation, and the relative abundance of most mineral samples was higher than 10%. Both chemoheterotrophy and aerobic chemoheterotrophy were considered broad ecosystem functions, and performed by most microorganisms [70], such as *Acidobacteria*, *Proteobacteria*, and *Verrucomicrobia* [71]. In our study, chemoheterotrophy was mostly related to *Euryarchaeota*, *Proteobacteria*, *Acidobacteria*, and *Firmicutes*. The abundant chemoheterotrophy in hot springs suggested that the majority of mineral-associated microorganisms cannot fix carbon and have to oxidate organic compounds to obtain energy and carbon [72]. Whitman and collaborators revealed that initial colonizers of fresh minerals largely contribute to stabilizing organic carbon by their necro mass that becomes mineral associated [24], and several studies also showed that the minerals structure was beneficial for the organic matter absorption [8, 15, 69]. We speculated that the addition of minerals in the microcosms increases the absorption of organic matter that benefits heterotrophic microorganisms. Additionally, a large number of knallgas bacteria and dark hydrogen oxidation in GMQ_group_1 (surrounding sediment, biotite, K-feldspar, and muscovite) were defined as the group of hydrogen-oxidizing bacteria (mostly related to *Hydrogenobacter*), which are energy-efficient in carbon dioxide fixation [73]. Colonization of these chemolithotrophs may also facilitate the growth of heterotrophs on the mineral surfaces.

In addition, some microbial ecological functions identified from minerals-associated taxa exhibited different relative abundances in the two hot springs. Moreover, the dominant functional groups enriched by the same mineral in the two different hot springs were not the same. In GMQ, as an indispensable part of the nitrogen cycle, the high enrichment of the denitrification pathway (including denitrification, nitrate denitrification, nitrite denitrification, and nitrous oxide denitrification) in aragonite incubation was dominated by *Rhodobacteraceae* (order *Rhodobacterales*) [74] and promoted the nitrogen cycles (Figure S1). The high proportion of anoxygenic photoautotrophy Fe oxidizing and anoxygenic photoautotrophy H_2_ oxidizing in hematite was attributed to *Rhodopseudomonas palustris*, which has the capability to fix CO_2_ as biomass [75]. Contrastingly, the functional groups of bacteria associated with the sulfate respiration and respiration of sulfur compounds increased significantly in the aragonite microcosm in the WGT. Our results were coincident with previous reports [76, 77], where sulfur and sulfate respiration were attributed to the dominance of Deltaproteobacteria. For aragonite microcosm in WGT, the respiration of sulfur compounds bacteria groups was related to the family *Desulfobulbaceae* (well known as cable bacteria), and the functional groups of sulfate respiration were mainly related to genus *Dissulfurimicrobium* (family: *Desulfobulbaceae*) and *Desulfomonile* (family: *Syntrophaceae*). Documentary evidence shows that sulfate-reducing bacteria (SRB) were generally in a neutral environment (the optimum pH is 7 - 8) with few isolated or enriched below pH 5 [78–81], while the bulk water pH of the WGT source (≤ 4.0) exceeds the reported growth pH range for *Dissulfurimicrobium* [82] and *Desulfomonile* [83, 84]. Previous studies have confirmed that carbonate minerals could neutralize acidity produced by neutrophilic bacteria [19]. However, Lin et al. verified that the metabolic rate of the sulfate-reducing bacteria (*Desulfovibrio bizertensis*) decreased substantially at above 80% carbonate minerals. [85]. Notably, on the microscopic scale, microbial compositions are more closely related to the microenvironment in which they live rather than the macroenvironment [16, 63]. We speculated that the buffering capability of aragonite provides SRB with a stable and suitable living environment. Hence, minerals could provide advantageous surface properties or microhabitats to protect microbes against extrinsic, harsh environmental conditions. In addition, the methanol/methane oxidation bacteria enriched in aragonite were mostly *Methylophilaceae*, and the remarkable sulfate respiration process in aragonite is coupled with methanol oxidation to promote the metabolism of the carbon cycle.

## Conclusion

The present field experiment study showed that the physicochemical properties of minerals affect the taxonomic microbial community in hot springs. Statistical analysis also revealed that minerals play an important role in shaping the alpha and beta diversity of hot spring microbial communities. In parallel, the enrichment of the metabolic function of chemoheterotrophy, followed by carbon, nitrogen, and sulfur cycling in minerals-associated communities, is potentially caused by the high organic matter adsorption ability of these fresh minerals. The high abundance of neutrophilic *Desulfobulbaceae* and sulfur-reducing functions within the aragonite microcosm in WGT suggested pH buffering of the mineral in the acidic spring. Our findings provide a basis to explore the impact of minerals on the taxonomic and functional structures of the microbial community in hot springs and also provide clues for the biogeochemical element cycle in extreme environments. Further studies are required to clarify the effects of minerals on the gene expression and metabolic functions of the thermophiles in hot springs.

## Electronic supplementary material

Below is the link to the electronic supplementary material.

Additional file 1. **Figure S1**: Heatmap of the FAPROTAX analysis among minerals and surrounding sediment samples in GMQ and WGT springs. **Table S1**: Metabolic diversity of all samples based on the FAPROTAX analysis.

## Data Availability

Sequence data have been deposited in the Genome Sequence Archive in the National Genomics Data Center, Beijing Institute of Genomics (China National Center for Bioinformation), Chinese Academy of Sciences, under accession number (CRA009208) that are publicly accessible at https://bigd.big.ac.cn/gsa.
