# Supplementary material for: Effects of Mineral on Taxonomic and Functional Structures of Microbial Community in Tengchong Hot Springs via in-situ cultivation
Source: Environ Microbiome. 2023 Mar 22;18:22. doi: 10.1186/s40793-023-00481-1 (PMC10035157; doi:10.1186/s40793-023-00481-1)
Supplement: Supplementary file 1 — Additional file 1. Figure S1: Heatmap of the FAPROTAX analysis among minerals and surrounding sediment samples in GMQ and WGT springs. Table S1: Metabolic diversity of all samples based on the FAPROTAX analysis. [file 40793_2023_481_MOESM1_ESM.docx]

Effects of Mineral on Taxonomic and Functional Structures of Microbial Community in Tengchong Hot springs via *in-situ* cultivation

Fangru Li^1^, Weiguo Hou^1*^, Shang Wang^2^, Yidi Zhang^1^, Qing He^2^, Wenhui Zhang^1^, Hailiang Dong^1^

^1^ Center for Geomicrobiology and Biogeochemistry Research, State Key Laboratory of Biology and Environmental Geology, China University of Geosciences, Beijing 100083, China

^2^ CAS Key Laboratory of Environmental Biotechnology, Research Center for Eco-Environmental Sciences, Chinese Academy of Sciences (CAS), Beijing, 100085, China

***Corresponding author:**

Weiguo Hou: [weiguohou@cugb.edu.cn](mailto:weiguohou@cugb.edu.cn)

# Supplementary Material


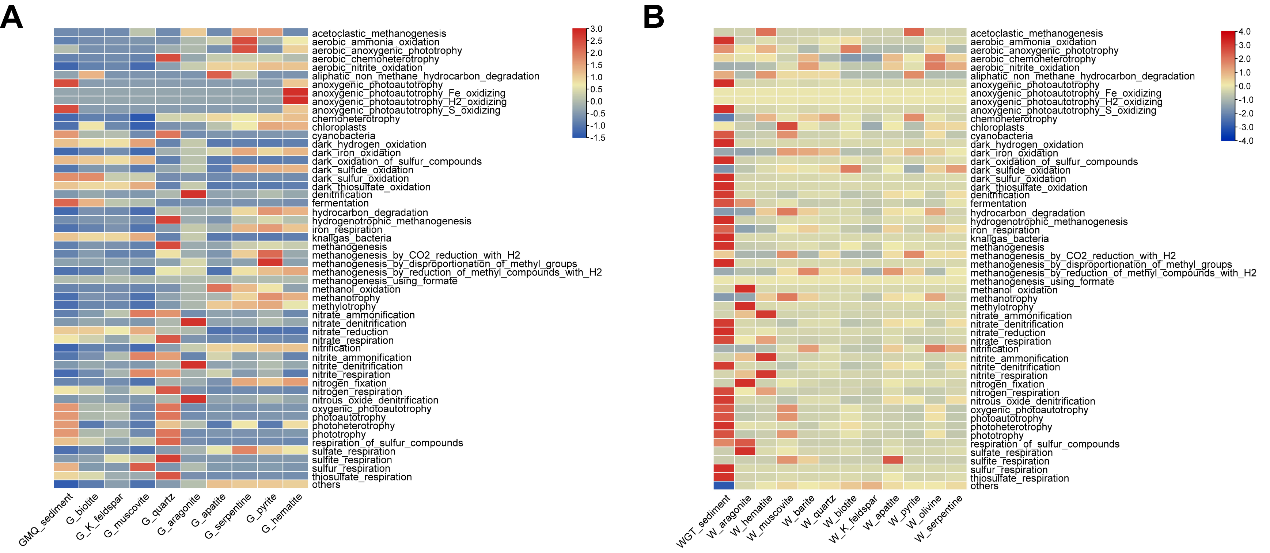


**Figure S1.** Heatmap of the FAPROTAX analysis among minerals and surrounding sediment samples in GMQ and WGT springs.

**Table S1.** Metabolic diversity of all samples based on the FAPROTAX analysis

|  | | Shannon | Inv_simpson | Pielou_evenness |
| --- | --- | --- | --- | --- |
| GMQ | sediment | 2.56 | 10.70 | 0.69 |
|  | biotite | 2.46 | 9.13 | 0.65 |
|  | K_feldspar | 2.22 | 6.34 | 0.59 |
|  | muscovite | 2.29 | 8.07 | 0.62 |
|  | quartz | 2.34 | 6.90 | 0.61 |
|  | aragonite | 1.99 | 4.35 | 0.52 |
|  | apatite | 1.11 | 1.94 | 0.30 |
|  | serpentine | 1.30 | 2.09 | 0.34 |
|  | pyrite | 1.21 | 2.05 | 0.32 |
|  | hematite | 1.24 | 2.13 | 0.32 |
| WGT | sediment | 2.41 | 7.81 | 0.62 |
|  | aragonite | 1.40 | 2.24 | 0.37 |
|  | hematite | 1.13 | 1.90 | 0.30 |
|  | muscovite | 1.00 | 1.67 | 0.26 |
|  | barite | 1.04 | 1.84 | 0.28 |
|  | quartz | 0.92 | 1.72 | 0.24 |
|  | biotite | 0.82 | 1.53 | 0.22 |
|  | K_feldspar | 0.61 | 1.38 | 0.16 |
|  | apatite | 1.02 | 1.71 | 0.27 |
|  | pyrite | 0.89 | 1.76 | 0.23 |
|  | olivine | 1.09 | 1.85 | 0.29 |
|  | serpentine | 1.02 | 1.71 | 0.27 |
